# Case report of non-tracheal intubation—an alternative for postpneumonectomy patients undergoing contralateral pulmonary resection

**DOI:** 10.1186/s13019-023-02386-z

**Published:** 2023-10-10

**Authors:** Jingdan Deng, Zhiwen Zeng, Zizheng Zhang

**Affiliations:** 1 Department of Anesthesiology, Meizhou People’s Hospital, 514031 Meizhou City, Guangdong Province China; 2 Department of thoracic surgery, Meizhou People’s Hospital, 514031 Meizhou City, Guangdong Province China

**Keywords:** One-lung patient, Video-assisted thoracoscopic surgery, Lung cancer, Laryngeal mask airway, Non-tracheal intubation

## Abstract

**Background:**

Surgery on the contralateral or other lungs after pneumonectomy on one side is highly challenging and complex. It is critical to creating conditions for fluent surgical maneuvers while ensuring adequate ventilation for a patient during such an operation in the same chest cavity that appears incompatible.

**Case presentation:**

We have reported herein the case of a patient who, following a left pneumonectomy, underwent a right upper pulmonary nodule wedge resection via video-assisted thoracoscopic surgery without requiring endotracheal intubation. We managed ventilation with a laryngeal mask airway under general anesthesia combined with a thoracic epidural block. The diseased lobe collapsed well for the surgical procedure during VATS without hypoxia, after which the resection was safely performed.

**Conclusions:**

Non-tracheal intubation anesthesia can be a potentially attractive alternative for patients undergoing contralateral pulmonary resection after pneumonectomy.

**Supplementary Information:**

The online version contains supplementary material available at 10.1186/s13019-023-02386-z.

## Background

Single-lung excision induces significant physiological changes in patients [1]. New lesions may be detected in a contralateral lung after pneumonectomy. Airway management in this type of surgical treatment during anesthesia is crucial and complicated, considering the procedure is uncommon and infrequently performed [2]. For patients’ safety and to accommodate thoracoscopic operation with the collapse of the lung in the region of surgery, oxygen must undoubtedly be delivered without interruptions for a long time while simultaneously allowing breathing movement of the lobes in the non-surgical regions. It has been reported that, during surgery, intraoperative pauses in breathing are extremely common, and broncho-occluders (BB), double-lumen tracheal catheters (DLT), high-frequency jet ventilation (HFJV), and extracorporeal membrane oxygenation (ECMO) are all effective in resolving these issues.

Our research demonstrated that a relatively simple approach involving non-tracheal intubation with a laryngeal mask airway (LMA) could similarly assure patient safety and the effective execution of surgery.

### Case presentation

**Fig. 1:**
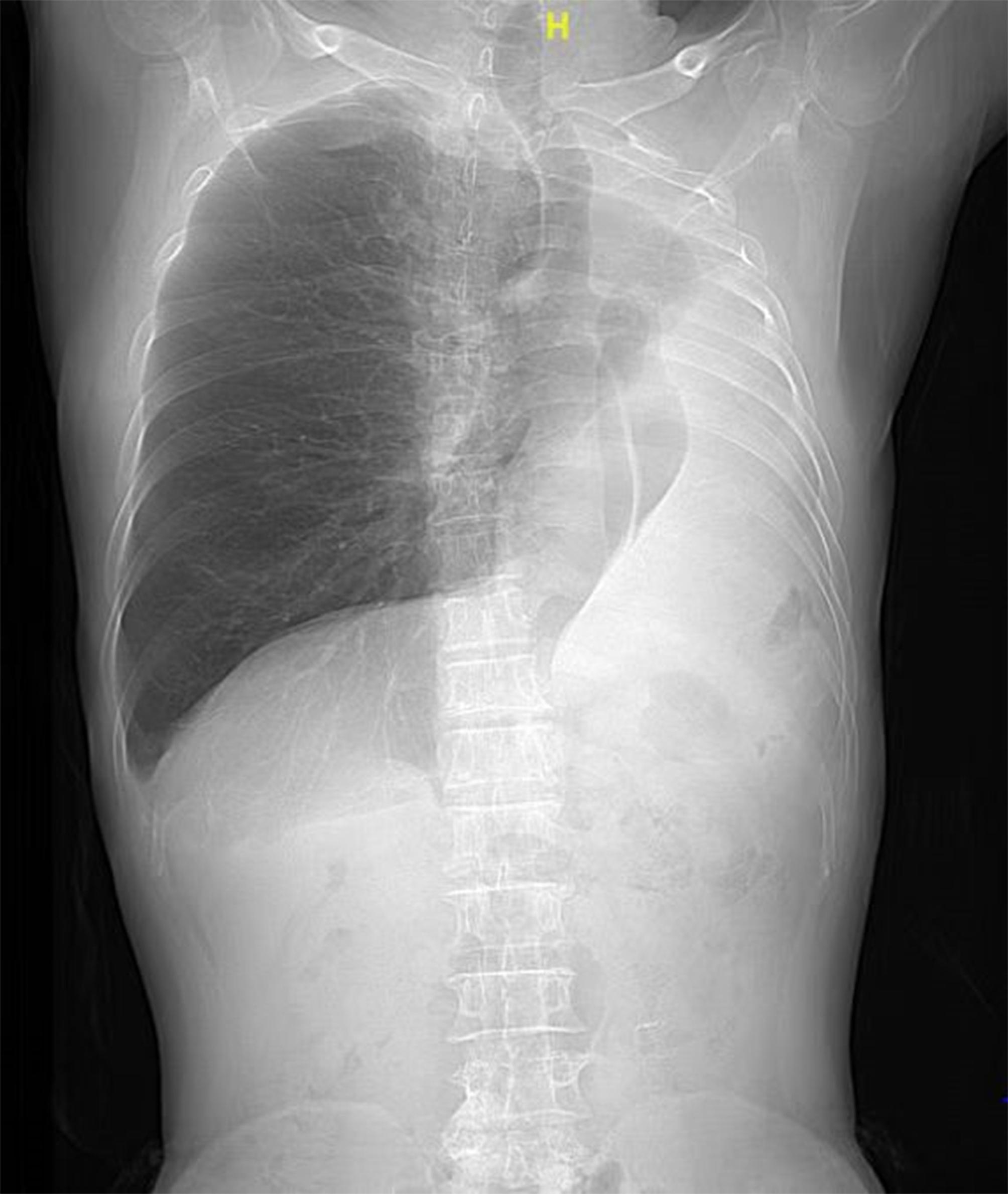
The patient had a left pneumonectomy surgery earlier

**Fig. 2:**
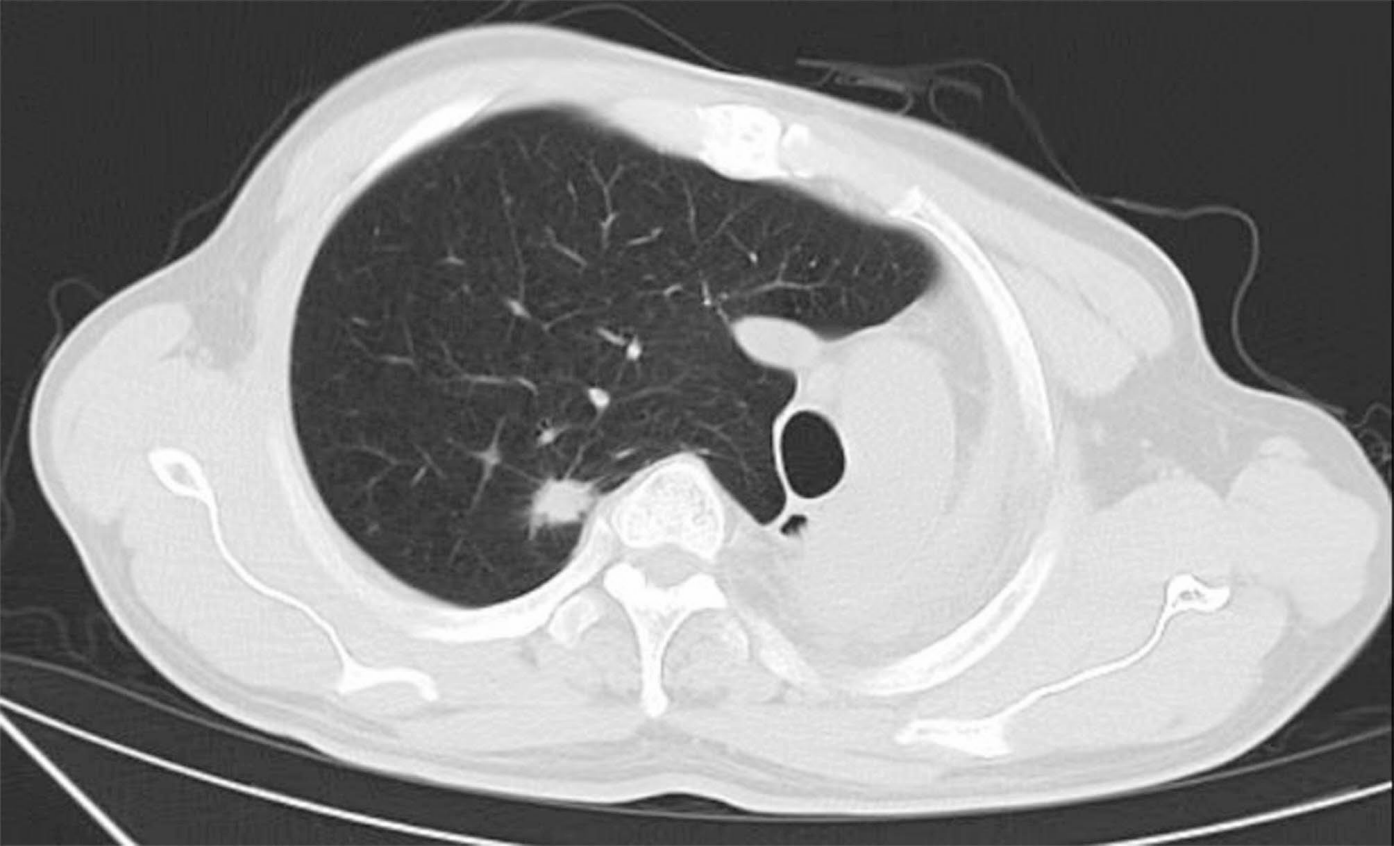
CT revealed a nodule in his right upper lobe

After undergoing a left pneumonectomy for moderately differentiated squamous cell carcinoma at the tracheal opening in the left upper and lower lobes more than four years ago, a 67-year-old man (weight: 53.4 kg; height: 155 cm; body mass index [BMI]: 22.22) was admitted to the hospital for a right upper-lobe nodule with a spiculated sign, as confirmed by repeated computed tomography (CT) imaging a week before admission (Figs. 1 and 2).

He could ascend three stories without stopping. His maximum voluntary breathing was 73.95 L/min, vital capacity was 2.62 L, forced expiratory volume in one second (FEV_1_) was 1.70 L, and the ratio of FEV1 to FVC was 65.05%. The arterial blood gas test revealed a PaCO_2_ level of 46.3 mmHg, PO_2_ level of 82.6 mmHg, SaO_2_ level of 96.9%, and FiO_2_ level of 21%. Transthoracic echocardiography revealed no significant abnormalities, such as right heart failure or pulmonary hypertension, except for mild aortic and tricuspid regurgitation.

The CT revealed a preference for a primary tumor, and the interval of more than four years since the last surgical treatment was considered a chronotropic tumor based on the ACCP guidelines [3]. In addition, the patient did not agree to undergo radiation or chemotherapy. Therefore, after the assessment that the patient had an adequate pulmonary reserve, a surgical wedge resection procedure was developed to maintain his optimal lung ventilation function.

### Anesthetic management and surgical procedure

**Fig. 3:**
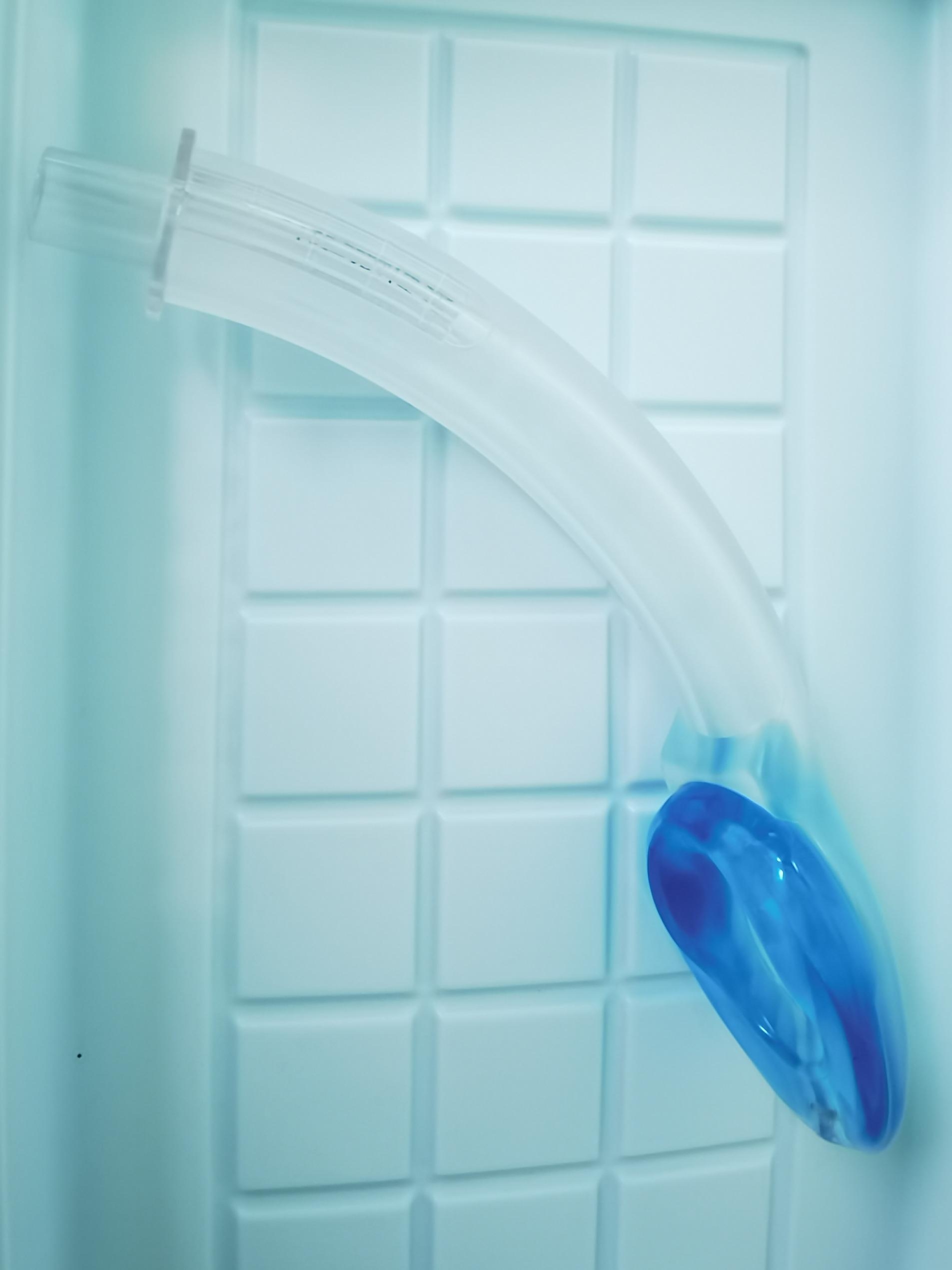
The GMA laryngeal mask

**Fig. 4:**
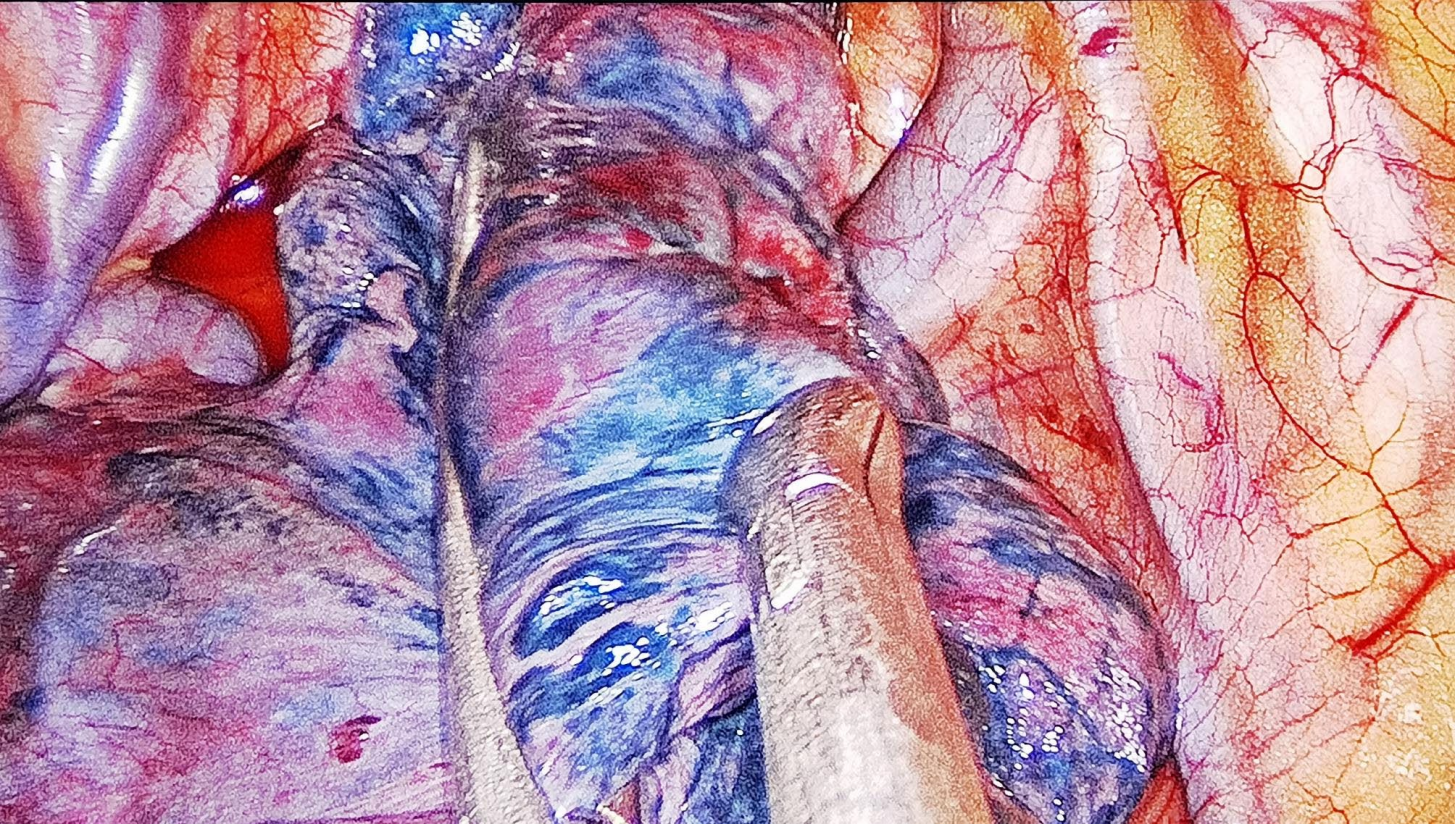
The right-upper lung collapsed during the surgery

Electrocardiography, percutaneous oxygen saturation (SpO_2_) measurement, noninvasive blood pressure, blood pressure of the left radial artery monitoring, end-tidal carbon dioxide monitoring, Bi-spectral Index (BIS), and body temperature monitoring were all performed as a part of the intraoperative monitoring. The sixth and seventh interspaces of the thoracic vertebra were utilized to insert an epidural catheter. Before the surgery, the patient was administered an epidural catheter of a 9-mL mixture of 0.375% ropivacaine and 1% lidocaine. Then, 10 µg of sufentanil, 25 µg of dexmedetomidine, and target-controlled infusion (TCI) of propofol were used to induce general anesthesia, which was then maintained with a TCI of propofol at an effect-site concentration (Ce) of 2–3 µg/mL and a tiny amount of remifentanil at the rate of 0.01–0.02 µg·kg^− 1^·min^− 1^. During the surgery, the BIS index was controlled at 40–60. No skeletal muscle relaxant was used. When the patient was asleep, a glottis mask airway (GMA) of the size of 4 (Medan Medical Device Co., Ltd., Tianjin, China), a type of LMA, was inserted without any untoward incident (Fig. 3). In addition, the glottic deviation was observed during LMA localization with fiberoptic bronchoscopy because of mediastinal displacement due to left lung resection. However, no detectable gas escape was detected when the breathing bag was compressed. The spontaneous breath was preserved at the beginning. After entering the thoracic cavity, the surgeon sprayed 5 mL of 2% lidocaine into the visceral pleural and lung surface. The right vagus nerve was then blocked by injecting a 2.5-mL mixture of 1% lidocaine and 0.375% ropivacaine. The patient could breathe spontaneously and undergo a successful operation due to artificial pneumothorax. The surgeon squeezed air by compressing the patient’s right upper lung. When this lobe collapsed, the upper margin of the thoracic cavity became visible. Its surface color changed to faint purple (Fig. 4), although both the right middle and the right lower lungs continued to dilate. The intermittent analyses of arterial blood gas data were performed (Table 1). About 17 min after the surgery began, PaCO_2_ rose from 53.8 mmHg to 91.7 mmHg, while the PaO_2_ level reduced to 75.2 mmHg. The patient was then placed on mechanical ventilation; his tidal volume-control settings were set to 200 mL at the respiratory rate of 15 bpm in the volume-control ventilation mode. The inspiratory/expiratory ratio was 1:2, and the fraction of inspired oxygen (FiO_2_) was set to 1.0 without using any positive end-expiratory pressure (PEEP). The patient’s actual airway pressure was maintained at 7 cm of H_2_O.

**Table 1:** Blood gas analysis results at different time points during anesthesia

	Before the operation	Spontaneous breath	Mechanical ventilation	After the operation (the LMA was removed)
Time (am)	9:15	9:47	10:29	10:55
PaO_2_	250.3	98.2	272.7	126.8
PaCO_2_	53.8	91.7	65.2	51.8
SaO_2_	99.9	93.5	99.9	98.4
pH	7.26	7.1	7.19	7.26
BE(B)	-2.9	-2.9	-3.7	-3.4
BEecf	3.3	1.5	-3.5	-4.0
HCO_3_	24.1	28.4	24.9	23.3
HCO_3_std	22.0	21.9	21.3	21.6
TCO_2_	26	31	27	25
FIO_2_	100	100	80	40
Lac	1.0	0.9	0.5	0.7
Glu	4.95	4.04	4.92	4.84

**Fig. 5:**
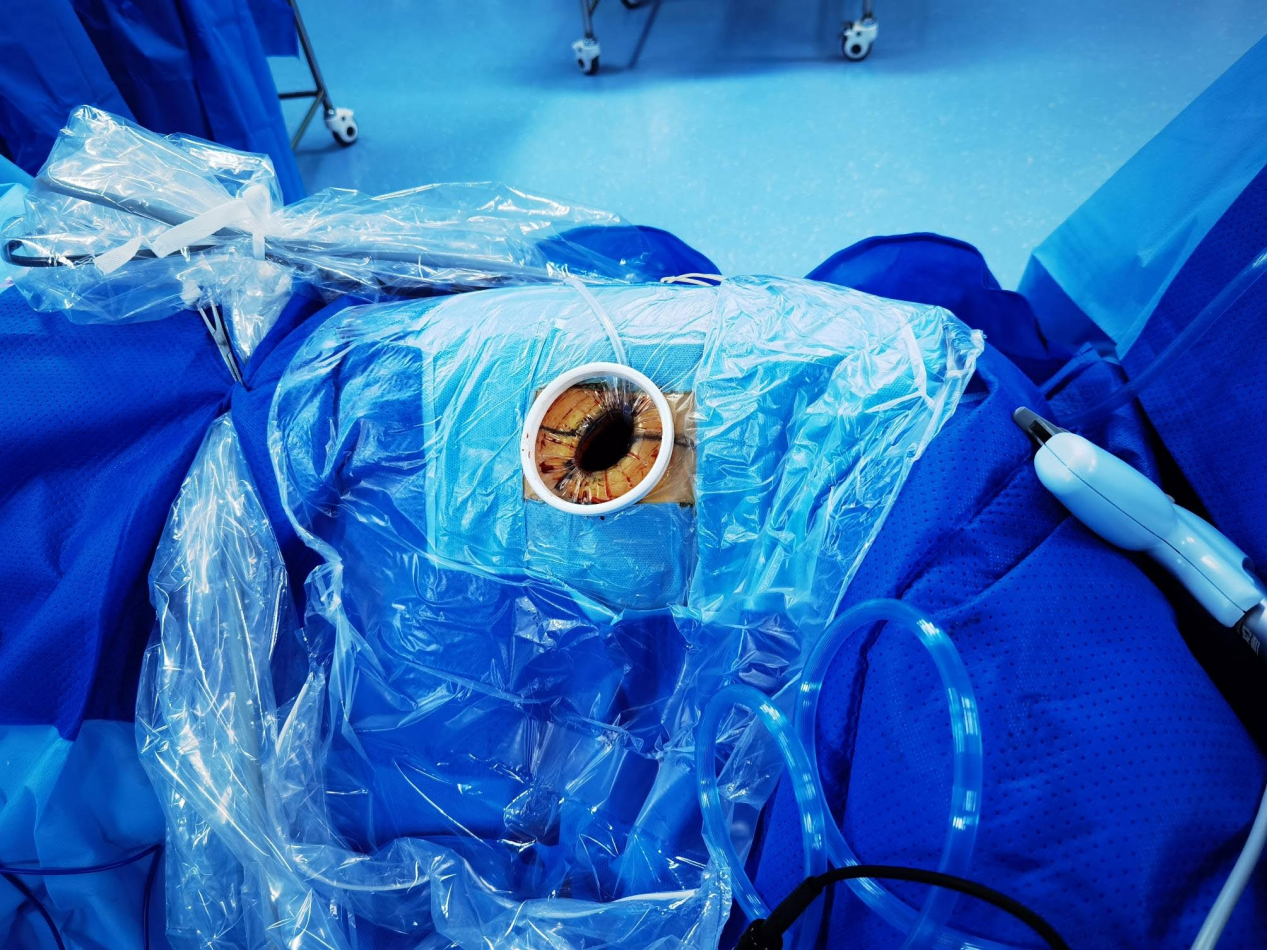
The 3-cm-long incision of the single-port VATS

During the surgery, the SpO_2_ level was maintained at > 93%, ensuring adequate oxygenation of the tissues. The patient’s blood pressure and heart rate remained constant during the phenylephrine intravenous pump-assisted, video-assisted thoracoscopic surgery (VATS). Consequently, the surgeon could undergo a wedge resection of the right upper lobe using powered compacted articulating endoscopic liner cutters (Echelon Flex™ 45, Johnson & Johnson Co., Ltd., New Jersey, USA) and lymph node sampling by VATS with a single incision of 3-cm long without any interruption (Fig. 5). At 20 min after the start of the surgery (ASOS), a fraction of inspired oxygen changed to 0.9, which changed to 0.8 at 50 min ASOS. Neither a cough nor a mediastinal swing manifested themselves throughout the surgery. No bubble was observed with the lung incision under water in the air-leak test. A chest drain tube was placed for drainage. At the end of the surgery, all the lobes were fully inflated on the video thoracoscopy. The duration of the surgery was roughly 75 min. In addition, the LMA was removed from the patient’s body 3 min after the operation. His postoperative pain was managed through patient-controlled epidural analgesia consisting of 3.125 mg/h ropivacaine and 0.521 µg/h sufentanil. No postoperative complications were developed. The pain score was 3 out of 10 on movement or coughing. The earliest postoperative chest X-ray revealed no evidence of effusion or gas in the chest cavity. The patient spent two nights in the hospital after surgery. The tube draining fluid from the chest was removed before the patient was discharged. The pathology results from the surgical tissues confirmed a diagnosis of lung adenocarcinoma, and the immunohistochemistry stain was positive, demonstrating the presence of thyroid transcription factor 1. The patient returned to the outpatient clinic for a follow-up with no discomfort a month later. His CT scan revealed no new abnormalities. During a telephone interview six months after surgery, the patient reported no significant decrease in activity tolerance relative to before the second surgery.

## Discussion and conclusions

Patients with a pneumonectomy may require other surgical procedures to treat various illnesses. About 3% of such patients reportedly experienced subsequent contralateral non-small cell lung cancer, and only 16% had surgical resection alone. However, these resection patients had higher 1-year and 3-year survival compared to those patients who received radiation and no treatment [4].

Right upper-lobe lesions may be treated using a single-lumen tracheal tube (SLT) intubating into the right bronchus intermedius. Another method for these patients to maintain ventilation during surgery is with a bronchial blocker (BB), which can selectively block any lobe without any restrictions [5–7]. Kawamoto and Walsh, with their respective teams, achieved a collapse of the right inferior lobe or the left inferior lobe using BB for one-lung patients. Double-lumen endobronchial tubes were used for this surgical purpose, considering a classic lung isolation technique. A 32-Fr left-sided double-lumen tube was used by Venkitaraman B, who inserted it into the right intermediate bronchus with the opening of the right catheter precisely facing toward the orifice of the upper lobe bronchus [8]. To make a 32-Fr left-sided DLT more manageable for short Asian women, Gu Y snipped off the tube’s tip [9]. In all their reports of using DLT, the surgical sites were a segment of the right upper lobe. DLT appeared to fail at its ventilation purpose when the lesion was located in the middle of the inferior lobe. Moreover, HFJV used a single-lumen endotracheal tube as an option for these patients. Ishiyama [10] reported a case wherein a suitable surgical condition was acquired with an increasing PaCO_2_. However, all these techniques require complicated positioning skills or even a particular airway appliance. SLT and DLT were only used in patients with upper lobe pulmonary lesions and not in lower lobe and middle lobe lesions. In addition, the premise of the use of these two types of catheter is that the diameter and length of the catheter must match the length and inner diameter of the patient’s bronchus and the position of the opening of the upper lobe, along with improving the catheter according to the actual situation before application. Although the application of BB is not limited to upper lobe lesions, it must be ensured that the occlusive device has sufficient length to occlude the distal bronchus. Moreover, BB is difficult to operate and is associated with a specific failure rate. On the other hand, HFJV is limited by the fear of barotrauma [11].

Terzi performed ECMO via femoral vessels during these surgeries for three one-lung patients [12]. However, in recent years, ECMO has rarely been used as the first choice and primarily served as an alternative backup in most cases. Although it allows complete lung collapse to facilitate the necessary surgical procedure, its application incurs a high risk of hemorrhage in the operating field with anticoagulation. It should be conducted by personnel with sufficient experience and specific sets of high-cost. Except for the risk of bleeding, other complications include hemolysis, obstruction of the artery after intubation, and limb ischemia, among others [13].

Among all the techniques of one-lung ventilation known as a classic tool, DLT is the first choice, followed by BB. The third one is the insertion of SLT into the contralateral bronchus [14]. Breathing suspension is limited to short time procedures. Increasing the number of breathing pauses inconveniences the surgeon and fails to realize the continuity of surgical exposure for crucial steps that require long-time procedures, such as hemostasis, which involves potential risks. As for postpneumonectomy patients undergoing contralateral chest surgery, no order of choice is recommended. The best possible solution remains debatable.

Due to technological advances, non-tracheal intubation with an LMA has emerged as a novel method of one-lung ventilation for managing the airway in VATS. The patient recovers quickly, needs less time on artificial ventilation, and experiences fewer side effects of muscle relaxants [15]. Residual paralysis brought by muscle relaxants may complicate the perioperative management of these one-lung patients because even minor residual paralysis may have clinical consequences. Pulmonary muscles’ function is impaired, respiratory control is affected, and coordination of the pharyngeal muscles is somewhat harmed [16]. For these reasons, the non-intubated approach can be the option for patients with impaired respiratory function, such as patients with severe COPD [17].

For this patient, we applied non-tracheal intubation with LMA without muscle relaxants. Compared with an endotracheal tube, LMA needs fewer opioids to maintain patient tolerance, which reduces the depression of spontaneous ventilation. A 3-cm uniportal surgical incision allows the right thoracic cavity to communicate with the outside air. The right upper lobe collapsed sufficiently under the surgeon’s compression. The remaining two lobes, the right-middle and the right-lower ones, could ventilate autonomously because they were not compressed. It is hence suggested that the upper limit of PaCO_2_ for intervention in non-tracheal intubation is 80 mmHg [15]. However, the patient did not show any significant fluctuations in PO_2_ level and circulation under intensive monitoring, and the patient did not have contraindications for permissive hypercapnia. Therefore, to ensure his safety, we switched his breathing regime from spontaneous breathing to that of a short, mechanically assisted ventilation for intervention when his PaCO_2_ level became > 90 mmHg. With a laryngeal mask, breathing depression can be easily managed through manual or mechanical ventilation [18]. In addition to allowing for spontaneous ventilation with monitoring ventilator pressure and volumes, i-gel LMA can be replaced with SLT assisted with the guidance of FOB in the lateral or prone position, when necessary. Although spontaneous ventilation was not completely used throughout the surgery, the operation ran smoothly. In this non-intubated VATS, the movements of lung and mediastinal shift made surgical maneuvers more technically and patiently demanding [19]. The postoperative recovery went well, and the patient was satisfied with his progress. Non-tracheal intubation with LMA is an easy and convenient alternative to airway appliance positioning. Any lobe can collapse selectively to conduct the operation efficiently.

This patient received sufficient analgesia from thoracic epidural anesthesia, and there was no evidence of apparent mediastinal swing. Because only two lobes of the lungs are responsible for ventilation, safe oxygenation during the entire surgery cannot be guaranteed. When necessary, an agreement was reached on a discontinuous operation plan of using short apnea windows as the second choice with surgeons. In this case, however, there was no interruption of operation, and the patient’s oxygenation was maintained at > 75.2 mmHg. This approach, however, does not allow for lung isolation, which is necessary to prevent the lesioned lobe from soiling healthy lobes. This intraoperative airway management skill necessitates training and practice. In addition, pulmonary hypertension patients are contraindicated because they cannot tolerate increased PCO_2_ levels. Patients with a possibility of extensive pleural adhesion, such as those with a history of ipsilateral thoracic surgery, should be excluded [15].

With the development of minimally invasive radiotherapy techniques, stereotactic ablative radiation therapy, thermal ablation, and brachytherapy have become the other safe options for these patients [20]. However, while surgical treatment opportunities exist, some patients who undergo contralateral pulmonary resection after pneumonectomy may benefit from non-tracheal intubation airway management.

### Electronic supplementary material

Below is the link to the electronic supplementary material.

Supplementary Material 1

## Data Availability

The datasets used and/or analyzed during the current study are available from the corresponding author upon reasonable request.

## List of abbreviations

ASOS: after the start of surgery
BB: Bronchial blocker
BIS: Bi-spectral Index
BMI: Body mass index
Ce: Effect-site concentration
CT: Computed tomography
DLT: Double-lumen endobronchial tube
ECMO: Extracorporeal membrane oxygenation
FEV1: Forced expiratory volume in one second
FiO2: Fraction of inspired oxygen
FOB: Fiberoptic bronchoscope
FVC: Forced vital capacity
GMA: Glottis mask airway
HFJV: High-frequency jet ventilation
LMA: Laryngeal mask airway
MVV: Maximal voluntary ventilation
PaCO2: Pressure of carbon dioxide
PEEP: Positive end-expiratory pressure
PaO2: Arterial oxygen partial pressure
SaO2: blood oxygen saturation
SLT: Single-lumen tracheal tube
SpO2: Percutaneous oxygen saturation
TCI: Target-controlled infusion
TV: Tidal volume
VATS: Video-assisted thoracoscopic surgery
VC: Vital capacity
