# Supplementary material for: Case report of non-tracheal intubation—an alternative for postpneumonectomy patients undergoing contralateral pulmonary resection
Source: J Cardiothorac Surg. 2023 Oct 10;18:282. doi: 10.1186/s13019-023-02386-z (PMC10565958; doi:10.1186/s13019-023-02386-z)

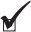

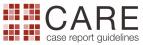

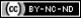
**CARE** **Checklist** **of** **information** **to** **include** **when** **writing** **a** **case** **report**


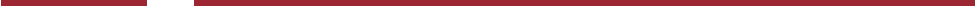


**Item** **Checklist** **item** **description**

**Reported** **on** **Line**

**Topic**


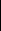


**Title**

**Key** **Words**

**Abstract**

**(no** **references)**

**Introduction** **Patient** **Information**

**Clinical** **Findings**

**Timeline**

**Diagnostic**

**Assessment**

**Therapeutic**

**Intervention**

**Follow-up** **and**

**Outcomes**

**Discussion**

**Patient** **Perspective** **Informed** **Consent**

**1** The diagnosis or intervention of primary focus followed by the words “case report” . . . . . . . . . . . . . . . . . . . . . . . . . . 1-2

**2** 2 to 5 key words that identify diagnoses or interventions in this case report, including "case report" . . . 33-34

**3a** Introduction: What is unique about this case and what does it add to the scientific literature? . . . . . . . . . . . . . . . . 22-26

**3b** Main symptoms and/or important clinical findings . . . . . . . . . . . . . . . . . . . . . . . . . . . . . . . . . . . . . . . . . . . . . . . . . . . . . . . 22-27

**3c** The main diagnoses, therapeutic interventions, and outcomes . . . . . . . . . . . . . . . . . . . . . . . . . . . . . . . . . . . . . . . . . . . 26-27

**3d** Conclusion—What is the main “take-away” lesson(s) from this case? . . . . . . . . . . . . . . . . . . . . . . . . . . . . . . . . . . . . . 29-30

**4** One or two paragraphs summarizing why this case is unique (**may** **include** **references**) . . . . . . . . . . . . 47-49

**5a** De-identified patient specific information. . . . . . . . . . . . . . . . . . . . . . . . . . . . . . . . . . . . . . . . . . . . . . . . . . . . 52-54

**5b** Primary concerns and symptoms of the patient. . . . . . . . . . . . . . . . . . . . . . . . . . . . . . . . . . . . . . . . . . . . . . . . . . . . . 54

**5c** Medical, family, and psycho-social history including relevant genetic information . . . . . . . . . . . . . . . . . 52-54

**5d** Relevant past interventions with outcomes . . . . . . . . . . . . . . . . . . . . . . . . . . . . . . . . . . . . . . . . . . . . . . . . . . . . . . . . 52

**6** Describe significant physical examination (PE) and important clinical findings. . . . . . . . . . . . . . . . . . . . . . . 52-61

**7** Historical and current information from this episode of care organized as a timeline . . . . . . . . . . . . . . . 52-61

**8a** Diagnostic testing (such as PE, laboratory testing, imaging, surveys). . . . . . . . . . . . . . . . . . . . . . . . . . . . . . . 54-55

**8b** Diagnostic challenges (such as access to testing, financial, or cultural) . . . . . . . . . . . . . . . . . . . . . . . . . . . . . no

**8c** Diagnosis (including other diagnoses considered) . . . . . . . . . . . . . . . . . . . . . . . . . . . . . . . . . . . . . . . . . . . . . . . . . 53

**8d** Prognosis (such as staging in oncology) where applicable . . . . . . . . . . . . . . . . . . . . . . . . . . . . . . . . . . . . . . . . . no

**9a** Types of therapeutic intervention (such as pharmacologic, surgical, preventive, self-care) . . . . . . . . . . . . . . . . . . . . 64-95, 99-100

**9b** Administration of therapeutic intervention (such as dosage, strength, duration) . . . . . . . . . . . . . . . . . . . . . . . . . . . . . 64-116

**9c** Changes in therapeutic intervention (with rationale) . . . . . . . . . . . . . . . . . . . . . . . . . . . . . . . . . . . . . . . . . . . . . . . . . . . . 64-110

**10a** Clinician and patient-assessed outcomes (if available) . . . . . . . . . . . . . . . . . . . . . . .. . . . . . . . . . . . . . . . . . . . . . . . . . . . 111-115

**10b** Important follow-up diagnostic and other test results . . . . . . . . . . . . . . . . . . . . . . . . . . . . . . . . . . . . . . . . . . . . . . . . . . . . 112-115

**10c** Intervention adherence and tolerability (How was this assessed?) . . . . . . . . . . . . . . . . . . . . . . . . . . . . . . . . . . . . . . . . . 108-109

**10d** Adverse and unanticipated events . . . . . . . . . . . . . . . . . . . . . . . . . . . . . . . . . . . . . . . . . . . . . . . . . . . . . . . . . . . . . . . . . . . no

**11a** A scientific discussion of the strengths AND limitations associated with this case report . . . . . . . . . . . . . . . . . . . . . . . 159-161

**11b** Discussion of the relevant medical literature **with** **references**. . . . . . . . . . . . . . . . . . . . . . . . . . . . . . . . . . . . . . . . . . 119-147

**11c** The scientific rationale for any conclusions (including assessment of possible causes) . . . . . . . . . . . . . . . . . . . . . . . . 144-147

**11d** The primary “take-away” lessons of this case report (without references) in a one paragraph conclusion . . . . . . . 166-167

**12** The patient should share their perspective in one to two paragraphs on the treatment(s) they received . . . . . . . . 108-110

**13** Did the patient give informed consent? Please provide if requested . . . . . . . . . . . . . . . . . . . . . . . . . . . . . . . . . . . . . . **Yes** √
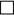
 **No**
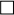

Supplement: Supplementary file 1 — Supplementary Material 1 [file 13019_2023_2386_MOESM1_ESM.docx]
